# AI-driven music intervention based on five-tone theory for anxiety: a preliminary pre-post feasibility study

**DOI:** 10.3389/fpsyg.2025.1669029

**Published:** 2025-12-10

**Authors:** Xu Rongrong, Li Jing

**Affiliations:** 1 School of Music and Dance, Xuchang University, Xuchang, China; 2 School of Arts, Zhejiang Normal University, Jinhua, China

**Keywords:** music therapy, five-tone theory, artificial intelligence, biofeedback, anxiety, autonomic nervous system, heart rate variability

## Abstract

Music therapy, as a pivotal non-pharmacological intervention, faces a critical challenge in designing personalized treatment protocols. Current clinical applications of the traditional Chinese five-tone theory to explore the relationship between music and emotion lack empirical validation and scalable implementation. This study integrates the five-tone theory with artificial intelligence (AI) to develop an AI-driven music modulation system based on real-time physiological feedback, aiming to examine its efficacy in alleviating anxiety symptoms and modulating the autonomic nervous system. A single-group pretest-posttest design was employed, with 40 university students exhibiting moderate anxiety (M_age = 21.5) enrolled as participants. Each underwent a 20-min intervention session. The system employed the Jue tone as the foundational pitch, with real-time dynamic modulation guided by heart rate variability (HRV) and electrodermal activity (EDA) data. The Hamilton Anxiety Rating Scale (HAMA), high-frequency HRV (HF-HRV), and skin conductance level (SCL) were employed in pre-post measurements. Post-intervention statistical analyses revealed a statistically significant reduction in HAMA scores (M_pre = 18.2 vs. M_post = 11.5, *p* < 0.001), accompanied by significant increases in HF-HRV and significant decreases in SCL (both *p* < 0.001). The preliminary findings suggest that the AI-powered music intervention integrating the five-tone theory with biofeedback mechanisms may have significant anxiolytic effects. This highlights the potential for incorporating traditional cultural wisdom into modern digital therapeutics and warrants further investigation through controlled trials.

## 1 Introduction

Music therapy has gained increasing recognition in the medical community as a non-pharmacological intervention for psychological disorders, including anxiety and depression (De Witte et al., 2021; Stegemann et al., 2019). It primarily involves modulation of key neural substrates such as the limbic system and prefrontal cortex, thereby regulating emotional processing, enhancing reward circuitry, and improving cognitive function (Koelsch, 2014). However, two critical challenges persist in clinical practices, which not only hinder the establishment of standardized protocols but also substantially constrain their widespread clinical adoption.

The first limitation lies in the implementation challenges of personalized application. Given the inherently subjective nature and cultural sensitivity of music therapy (McFerran and Saarikallio, 2014), conventional approaches rely predominantly on therapists’ empirical judgment and intuition for music selection, without a data-supported, systematically designed framework, consequently failing to achieve precise matching between musical interventions and individuals’ specific psychophysiological states (Olawade et al., 2024).

The second limitation involves the “disconnect between theory and empirical research.” Many traditional music therapy theories contain profound philosophical foundations, yet lack rigorous scientific validation. Taking traditional Chinese five-tone therapy as an example, this ancient system associates the five tones (gong, shang, jue, zhi, and yu) with specific organs (spleen, lung, liver, heart, and kidney) and emotional states (e.g., linking the jue tone with anger/anxiety) to form a unique theoretical framework (Matos et al., 2021). However, this millennia-honored system currently has yet to establish robust empirical evidence regarding its underlying mechanisms, clinical efficacy, and particularly the quantifiable relationships between specific acoustic frequencies and physiological states (Zhang and Lai, 2017; Zhang and Gao, 2022).

The rapid advancement of artificial intelligence (AI) and real-time biofeedback technologies presents a promising solution to these challenges. Leveraging such AI algorithms, it can produce highly complex and novel musical compositions, while wearable biosensors continuously monitor physiological indicators such as HRV and EDA, generating individualized health data streams. The integration of these two technologies enables the development of a dynamic interactive platform, transforming music creation from traditional passive approaches to active generation and personalized customization, thereby enhancing intervention outcomes and optimizing user experience.

This study endeavors to develop and conduct a preliminary evaluation of an AI-powered music therapy system incorporating the five-tone theory. As an initial step, it aims to advance interdisciplinary integration by assessing the system’s feasibility and its potential effects. As an exploratory study, the system seeks to investigate whether it can improve participants’ subjective anxiety and objective physiological stress responses through personalized music intervention protocols. Our exploration focuses on addressing the following core research questions:

*RQ*1: How does AI-generated music based on the five-tone theory influence anxiety scale scores?

*RQ*2: Can this intervention effectively modulate participants’ ANS activity, as measured by HRV and EDA?

## 2 Literature review

### 2.1 Psychophysiological foundations of music therapy

Music modulates emotional and physiological states by activating multiple brain regions. Research demonstrates its ability to regulate the limbic system, particularly the amygdala and hippocampus—key structures in emotional processing (Koelsch, 2014). Additionally, uplifting music triggers dopamine release, engaging the brain’s reward circuitry (Chanda and Levitin, 2013). From a physiological perspective, soothing music has been shown to reduce heart rate, blood pressure, and cortisol levels, thereby attenuating stress responses (Thoma et al., 2013; Jiang et al., 2016).

A critical psychoacoustic mechanism is rhythmic entrainment, the synchronization of internal neural oscillations with external acoustic rhythms (Thaut and Hoemberg, 2014). According to this theory, when individuals listen to music with specific tempi (e.g., 60–80 beats per minute), neural oscillations associated with relaxation (e.g., alpha waves) align with the musical rhythm (Ding et al., 2025). This synchronization shifts brain activity from a tense to a calmer state, serving as a core pathway for music’s regulatory effects on emotion and physiological arousal. The AI system in this study leverages this principle by dynamically adjusting musical parameters (e.g., tempo) via biofeedback to enhance entrainment effects.

### 2.2 Five-tone theory: a psychoacoustic model awaiting validation

Rooted in ancient Chinese music psychology, five-tone theory correlates the five tones (Gong, Shang, Jue, Zhi, and Yu) with five elements (earth, metal, wood, fire, and water), five visceral organs (spleen, liver, lung, heart, and kidney), and five emotional states (pensiveness, grief, anger, joy, and fear; see Table 1). It posits that specific musical modes can target corresponding organ systems and modulate associated emotions (Yang et al., 2021).

**Table 1:** The five tones in TCM: elemental, organ, emotional and functional correspondences.

Tone	Melodic style	Element	Emotion	Organ	Therapeutic Function
Jue	Lively, expansive	Wood	Anger (anxiety)	Liver, gallbladder	Soothes liver stagnation, calms nerves
Zhi	Energetic, vibrant	Fire	Joy	Heart, small intestine	Promotes yang, circulates blood
Gong	Solemn, steady	Earth	Pensiveness	Spleen, stomach	Strengthens spleen, stabilizes mood
Shang	Piercing, mournful	Metal	Grief	Lungs, large intestine	Tonifies qi, moistens lungs
Yu	Serene, flowing	Water	Fear	Kidney, bladder	Nourishes yin, tranquilizes mind

Source: Compiled from TCM classics.

It is critical to bridge the conceptual gap between TCM theory and modern psychophysiological measurement. In TCM, the “Jue” tone is linked to “liver qi” and the emotion of “anger/anxiety”. From a modern psychophysiological perspective, anxiety states are operationalized as a dysregulation of the autonomic nervous system (ANS), characterized by heightened sympathetic dominance (the “fight-or-flight” response) and reduced parasympathetic vagal tone (the “rest-and-digest” system). Therefore, this study hypothesizes that the TCM concept of “soothing the liver” (疏肝) to alleviate anxiety should manifest physiologically as a rebalancing of the ANS. We selected Heart Rate Variability (HF-HRV) as a robust marker of parasympathetic activity and Electrodermal Activity (SCL) as a marker of sympathetic arousal to serve as objective, quantifiable proxies for the targeted anxiolytic effect.

Despite its systematic framework, the theory’s foundational hypothesis—that specific sound frequencies “resonate” with target organs or their neural representations—remains philosophical. Recent studies have begun examining its mechanisms through modern medical and acoustic lenses (Liu et al., 2014). This study builds on that premise, using AI to generate music within theoretically specified frequency ranges and employing psychophysiological metrics to empirically test the ancient theory.

### 2.3 AI and biofeedback applications in music therapy

Emerging evidence suggests that combining AI with biofeedback enables an effective approach to personalized music therapy (Fedotchev et al., 2014; Li and Xiong, 2016; Rashmi and Shantala, 2024). AI algorithms can generate music based on users’ emotional labels or physiological signals, while biofeedback establishes a closed-loop system that continuously monitors intervention efficacy and dynamically adjusts musical parameters. For instance, when the system detects increased sympathetic nervous system activity—indicating heightened tension—it may automatically slow the musical tempo or simplify melodic complexity to guide the user back to a relaxed state (Şahin, 2025).

Current research has explored using AI to replicate established relaxation music styles (Kant and Sharma, 2023) or to develop more precise emotion-recognition models, such as those based on electroencephalogram (EEG) signals, laying the groundwork for truly responsive systems (Zhong et al., 2022; Jiao, 2025). However, few studies have employed AI as a “translation” and “validation” tool to systematically examine comprehensive traditional medical frameworks like the five-tone theory. The innovation of this study lies in leveraging AI as a bridge to transform this traditional qualitative theory into a quantifiable, testable, and personalized intervention, while empirically evaluating its efficacy.

## 3 Methods

### 3.1 Data source and participants

We recruited 40 university students (20 males, 20 females; mean age = 21.5 ± 2.2 years) through campus announcements and social media platforms.

Inclusion criteria: (1) Aged 18–28 years; (2) Hamilton Anxiety Rating Scale (HAMA) score ≥14, indicating at least mild anxiety (Ferguson et al., 2023; Hamilton, 1959); (3) No self-reported hearing impairments; (4) No systematic psychological or pharmacological treatments for anxiety in the past month.

Exclusion criteria: (1) History of severe mental disorders (e.g., schizophrenia, bipolar disorder); (2) Strong music aversion or amusia.

### 3.2 Research instruments

#### 3.2.1 AI music intervention system and procedure

We developed an AI-driven personalized music generation system with these core components and functions:

1. Theoretical framework: The algorithm incorporated five-tone theory, specifically utilizing the Jue tone (wood element, 329–391 Hz). Musical elements (scales, harmony, and melodic progression) were designed to embody traditional “liver-soothing and qi-regulating” properties.
2. Biofeedback module: The system connected via Bluetooth to wearable biosensors (Empatica E4 wristbands) for real-time HRV and EDA monitoring.
3. Closed-loop adjustment mechanism: The AI algorithm analyzed incoming physiological data every 30 s. Upon detecting elevated physiological arousal (e.g., decreased HF-HRV or increased SCL), it automatically modulated musical parameters—including tempo reduction (BPM), melodic simplification, and attenuation of high-frequency instrumentation—to promote relaxation. Conversely, it maintained or reversely adjusted these parameters when physiological indicators suggested optimal intervention efficacy, thereby sustaining therapeutic effectiveness.

##### 3.2.1.1 Generative algorithm and closed-loop logic

The AI generative model was a rule-based system designed to adhere strictly to the “Jue” tone framework. The generative algorithm ensured that melodic progressions prioritized the Jue (329–391 Hz) as the foundational pitch, while harmonic structures were constrained to modes traditionally considered “liver-soothing”.

The closed-loop adjustment mechanism, which analyzed data every 30 s, operated on specific triggers. A physiological arousal state was defined as a decrease in HF-HRV (e.g., >10% from baseline) or an increase in SCL (e.g., >0.1 μS from baseline). When such a trigger was detected, the AI algorithm automatically modulated the music in real-time. This modulation included: (1) reducing the tempo (BPM) by 5%, (2) simplifying the melodic complexity (e.g., reducing the note density per measure), and (3) attenuating the amplitude of higher-frequency instrumentation. To avoid abrupt discontinuities, all transitions between musical segments were smoothed using a 3-s crossfade.

#### 3.2.2 Measurement tools

For psychological assessment, we employed the Hamilton Anxiety Rating Scale (HAMA), a 14-item instrument. To ensure reliability and consistency, the HAMA was administered via a structured interview by trained research assistants (it was not used as a self-report measure). As a gold-standard measure for evaluating anxiety severity, the HAMA demonstrates well-established reliability and validity (Ferguson et al., 2023; Hamilton, 1959).

For physiological assessment, we derived two key autonomic nervous system indices from Empatica E4 wearable biosensor data:

Heart rate variability (HRV): We extracted the high-frequency component (HF-HRV, 0.15–0.4 Hz, unit: ms^2^), a well-validated marker of vagal tone that reflects parasympathetic nervous system tension (commonly termed the “rest-and-digest” system), with higher values indicating greater relaxation states (Maier et al., 1988; Shaffer and Ginsberg, 2017).

Electrodermal Activity (EDA): We quantified electrodermal activity (EDA) through tonic skin conductance level (SCL, unit: μS), a well-established psychophysiological marker of sympathetic nervous system activation (commonly termed the “fight-or-flight” response), with lower SCL values corresponding to reduced stress and physiological arousal (Shaffer and Ginsberg, 2017; Boucsein, 2012).

### 3.3 Study design

**Figure 1:**
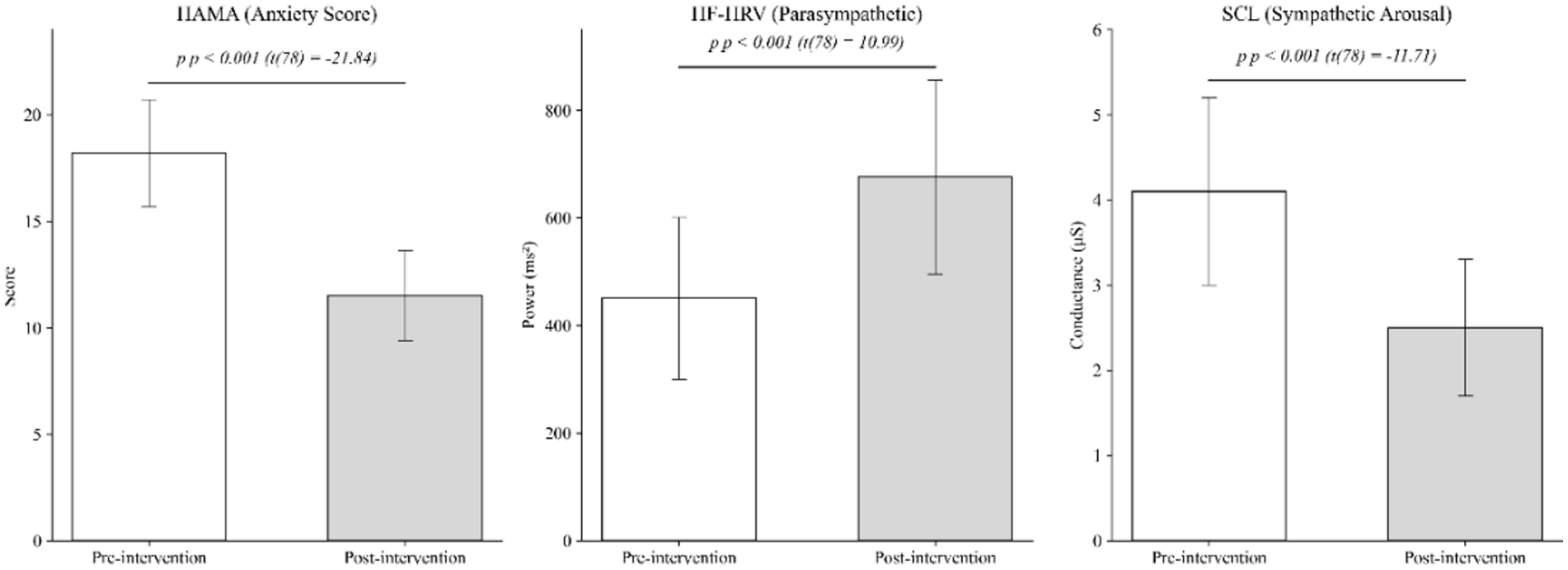
Interaction logic diagram of the AI-driven five-tone biofeedback music therapy system.

We implemented a single-group pre-test/post-test quasi-experimental design to evaluate the immediate effects of AI music intervention (see Figure 1 for study flowchart). The experimental protocol comprised the following steps:

1. Informed consent and ethical procedures: Participants received detailed explanations of study objectives, procedures, potential risks, and benefits before providing written informed consent;
2. Pretest: Trained research assistants administered the HAMA;
3. Baseline measurement: Participants wore Empatica E4 wristbands while sitting quietly for 5 min to establish physiological baselines;
4. Intervention: Participants received 20 min of AI-generated music through high-quality headphones;
5. Posttest: Immediate HAMA reassessment following intervention, with physiological data averaged from the final 5 min of the session serving as post-intervention measures.

### 3.4 Data analysis

All statistical analyses were performed using Python (v3.13) with the statsmodels library. To account for intra-individual variability from repeated measurements, as recommended, we employed linear mixed-effects models (LMM). For each of the three models, the outcome measure (HAMA score, HF-HRV, or SCL) was entered as the dependent variable. Time (pre-test vs. post-test) was entered as the fixed effect, and Participant was entered as a random effect (random intercept). Effect sizes were calculated using Cohen’s d. The significance threshold (*α*) was set at 0.05 for all statistical tests.

### 3.5 Ethical considerations

The study protocol was approved by the Institutional Review Board of the School of Law at Xuchang University. All participants provided *written* informed consent after receiving complete disclosures regarding study procedures, potential risks, and benefits. To ensure confidentiality, all collected data were anonymized and de-identified prior to analysis.

## 4 Results

The AI-driven music intervention demonstrated significant positive changes across both psychological and physiological measures.

### 4.1 Psychological outcomes

Participants demonstrated significant reductions in HAMA scores from pre-intervention (*M* = 18.2, SD = 2.5) to post-intervention (*M* = 11.5, SD = 2.1). The linear mixed-effects model confirmed this decrease was statistically significant (*t*(78) = −21.84, *p* < 0.001), with a large effect size (Cohen’s *d* = 1.95) (Boucsein, 2012), indicating substantial clinical improvement in subjective anxiety experiences following the intervention.

### 4.2 Physiological outcomes

**Figure 2:**
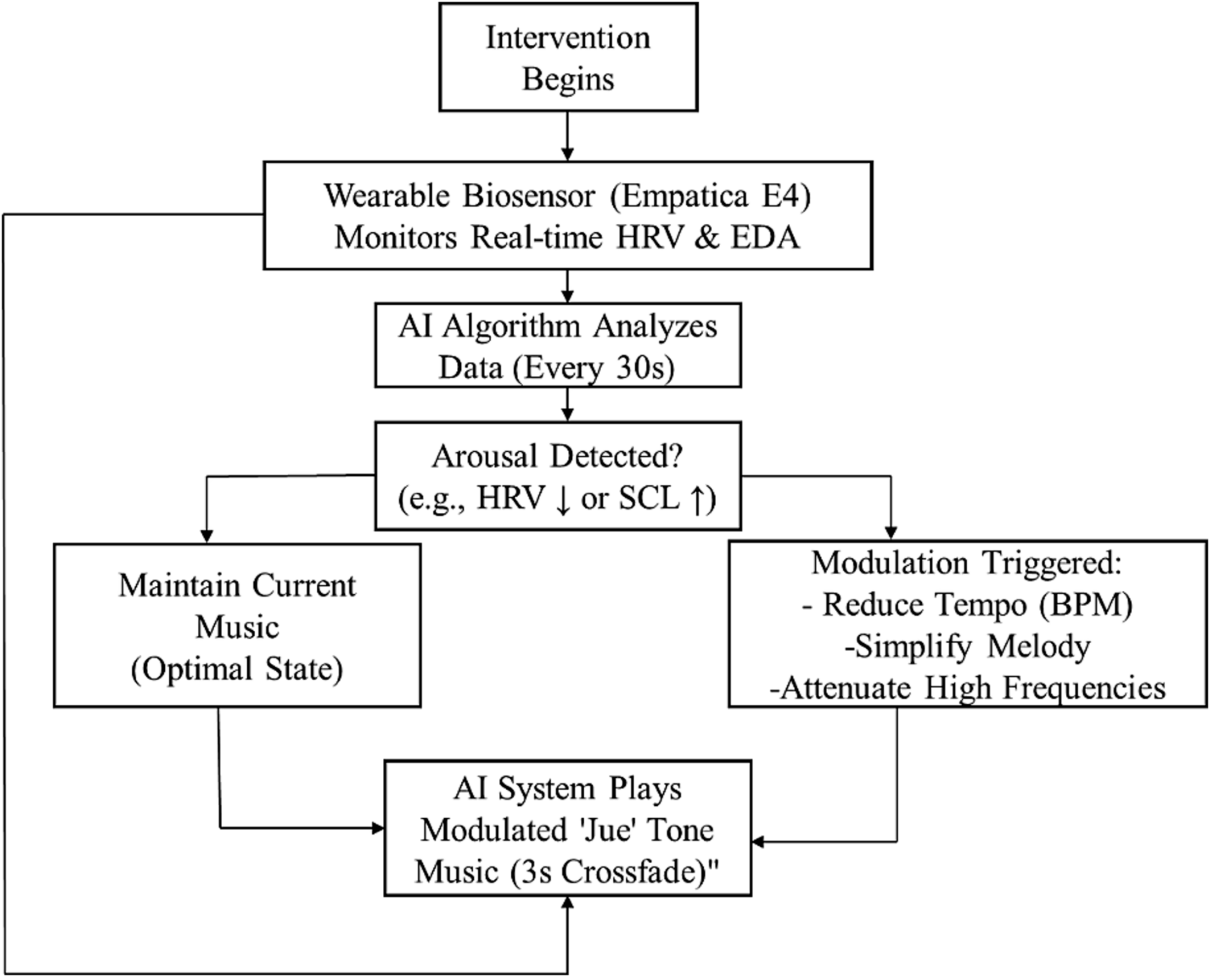
Pre-post comparisons of psychological and physiological metrics (*N* = 40).

Physiological data aligned with psychological reports (Figure 2). Post-intervention, participants exhibited a pronounced shift toward relaxation in autonomic nervous system (ANS) activity:

For parasympathetic nervous system activity (measured by HF-HRV), the mean values significantly increased from 450.5 ms^2^ (SD = 150.2) at baseline to 675.8 ms^2^ (SD = 180.4) post-intervention (t(78) = 10.99, *p* < 0.001).

For sympathetic arousal (measured by SCL), mean values decreased from 4.1 μS (SD = 1.1) at baseline to 2.5 μS (SD = 0.8) post-intervention (*t*(78) = −11.71, *p* < 0.001).

## 5 Conclusion and discussion

### 5.1 Key findings

This study examined the efficacy of an AI music intervention system integrating Traditional Chinese Five-Tone Theory with modern biofeedback technology. The results strongly support our primary hypothesis that this novel intervention can simultaneously alleviate anxiety both psychologically and physiologically in undergraduates with moderate anxiety. The significant reduction in HAMA scores (effect size d = 1.95) demonstrates both statistical and clinical significance, with improvement levels comparable to brief psychotherapy or pharmacological interventions (Cohen, 1988).

More importantly, the subjective improvements were corroborated by objective physiological data: substantial increases in HF-HRV and decreases in SCL indicate a clear physiological mechanism involving a beneficial shift in autonomic nervous system balance—from sympathetic (“fight-or-flight”) dominance to parasympathetic (“rest-and-digest”) predominance (Lu et al., 2021). This psychophysiological concordance provides preliminary evidence for the intervention’s potential efficacy beyond subjective reports, although placebo effects cannot be excluded by the current design. This finding represents the study’s primary contribution.

It is important to interpret the large effect size observed for HAMA scores (Cohen’s *d* = 1.95) with extreme caution. While encouraging, this value is likely inflated due to the single-group pre-post design, which does not control for factors such as expectancy, attention, or placebo effects. Such large effects are not uncommon in preliminary, uncontrolled intervention studies.

### 5.2 Theoretical implications

From the perspectives of music psychology and interdisciplinary research, this study carries multiple significant implications. First, it provides a preliminary quantitative exploration of the ancient five-tone theory’s modern applicability and potential scientific basis. The observed anxiolytic effects of Jue-tone music offer preliminary quantitative evidence for the traditional “liver-anger/anxiety-Jue” correspondence supporting the “acoustic resonance hypothesis” that specific musical structures or acoustic parameters may preferentially interact with particular physiological systems or their neural representations—a promising avenue for further scientific exploration.

Furthermore, this study demonstrates AI’s potential as a “computational bridge.” Our designed AI system translates an abstract, qualitative traditional medical theory into executable computer parameters (e.g., pitch patterns, tempo ranges, harmonic progressions) that can be modulated by physiological signals, thereby connecting conceptual traditional wisdom with measurable modern psychophysiology. This methodology opens new possibilities for examining other traditional medical theories (e.g., Indian Raga therapy) or psychoacoustic hypotheses (Thayer et al., 2012).

### 5.3 Practical implications

The practical implications of this research are substantial. This AI-driven, biofeedback-enhanced system represents a highly personalized, scalable, and non-invasive digital mental health intervention tool. Unlike conventional one-size-fits-all relaxation music playlists, it dynamically adjusts auditory experiences based on users’ real-time physiological states, potentially revolutionizing treatment adherence and efficacy. This technological paradigm holds significant application potential in three key areas:

1. As digital therapeutics (DTx), the system could develop into clinically validated prescription digital therapeutics for conditions like generalized anxiety disorder.
2. As a public mental health tool, it could be deployed on accessible platforms as a powerful, low-cost solution for daily stress management and emotion regulation, thereby contributing to population-level mental health improvement—an area where music therapy has demonstrated notable effects for depression and related conditions (Khare et al., 2024).
3. For enhancing traditional therapies, this technology could integrate with conventional psychotherapy or Traditional Chinese Medicine treatments, providing therapists with objective, real-time feedback to optimize treatment protocols.

### 5.4 Limitations and future directions

While the results are promising, this study has several significant limitations that must be acknowledged and addressed in future research.

The primary limitation is the single-group pre-post design, which is insufficient to establish causal efficacy. He observed changes cannot be definitively attributed to the AI-driven music intervention itself, as they could be the result of non-specific factors such as expectancy, simple attention from researchers (i.e., Hawthorne effects), or spontaneous fluctuations in anxiety. Therefore, the claims of this paper are exploratory and generative, not confirmatory. Future studies must employ rigorous randomized controlled trial (RCT) designs. As suggested, this should include (1) an active control group (e.g., listening to generic relaxation music or a sham intervention like white noise), and (2) a theory-specific control (e.g., non-adaptive Jue-tone music, or adaptive music based on a different tonal theory) to isolate the specific effects of the Five-Tone framework and the biofeedback loop.

The current study only examined immediate intervention effects, leaving the long-term outcomes unknown. Longitudinal follow-up studies are needed to determine whether repeated, prolonged interventions can produce sustained anxiety reduction and whether effects diminish over time. Additionally, the relatively homogeneous sample of university students limits the generalizability of findings. Future validation should include more diverse populations: clinically diagnosed anxiety patients, adults across different age groups, and individuals from varied cultural backgrounds (Aalbers et al., 2017; Witusik and Pietras, 2019).

In conclusion, this investigation focused exclusively on validating the Jue tone within the five-tone theory. Subsequent research should systematically test other theoretical postulates—such as using Zhi tone for alleviating low mood or Yu tone for fear reduction—while also examining combinatorial effects of different tones. This systematic approach will ultimately yield a more comprehensive and precise AI-powered personalized treatment model based on five-tone theory.

## Data Availability

The original contributions presented in the study are included in the article/supplementary material, further inquiries can be directed to the corresponding author.
